# Geographic Distribution and Expansion of Human Lyme Disease, United States

**DOI:** 10.3201/eid2108.141878

**Published:** 2015-08

**Affiliations:** Centers for Disease Control and Prevention, Fort Collins, Colorado, USA

**Keywords:** Lyme disease, *Borrelia burgdorferi*, epidemiology, vector-borne infections, zoonoses, United States, tickborne, surveillance, bacteria

## Abstract

Lyme disease occurs in specific geographic regions of the United States. We present a method for defining high-risk counties based on observed versus expected number of reported human Lyme disease cases. Applying this method to successive periods shows substantial geographic expansion of counties at high risk for Lyme disease.

Lyme disease is a multisystem tickborne zoonosis caused by infection with the spirochete *Borrelia burgdorferi* (*1*,*2*). Since 1991, state and territorial health departments have reported human Lyme disease cases to the Centers for Disease Control and Prevention through the National Notifiable Diseases Surveillance System. Most cases are reported from the northeastern, mid-Atlantic, and north-central states, although the number of jurisdictions that report a high number of cases has increased over time (*3*). To better quantify and track the geographic distribution of human Lyme disease, we developed a simple but robust method for defining counties where residents have a high risk of acquiring this disease.

## The Study

Counties with a high incidence of Lyme disease were identified by using SaTScan version 9.1.1(*4*). Numbers of confirmed Lyme disease cases reported at the county level during 1993–2012 were aggregated into 5-year intervals (1993–1997, 1998–2002, 2003–2007, 2008–2012) to minimize the influence of travel-associated cases and short-term changes in surveillance practices. Incidence was calculated by using each county’s average population at risk, which was estimated from US Census data for the midpoint of each period (i.e., 1995, 2000, 2005, and 2010). Identification of high-risk clusters was based on county incidence rates, with a maximum possible cluster size equal to 25% of the US population (minimum size was 1 county). County centroids were used as geographic reference for analyses. During the study period, 3 different surveillance case definitions were used (i.e., those established in 1991, 1996, and 2008) (*5*).

Relative risk (RR) was defined as the observed number of cases divided by the expected number of cases for a specific period and population size, and adjusted for differences in the population at risk across space (*4*). Calculations were based on a discrete Poisson probability distribution. RR was calculated for potential clusters and for individual counties within detected clusters. Statistical significance of possible clusters was determined by using likelihood ratio tests and standard Monte Carlo hypothesis testing (n = 999 replications) (*4*).

Because of the circular shape used in spatial scanning, not all counties within an identified high-risk cluster were necessarily characterized by high Lyme disease incidence. Some may have been included because they share a border with a county having high incidence. Ultimately, counties designated as high incidence were those within a defined, statistically significant high-risk spatial cluster (α = 0.05) and with a county-specific RR >2.0.

In each period, 2 major foci of largely contiguous counties met the high-incidence county designation: 1 in the northeastern United States and 1 in the north-central United States (Figure). During the first 5-year period (1993–1997), 69 counties were characterized as having high incidence of Lyme disease, including 4 isolated counties in the southeastern United States (Table; Figure). During the next period (1998–2002), 130 counties were characterized as having high incidence, and the 4 counties in the southeastern United States ceased to meet the criteria for this designation. During the third and fourth periods (2003–2007 and 2008–2012), 197 and 260 counties, respectively, were characterized as having high incidence (Table; Figure).

**Figure:**
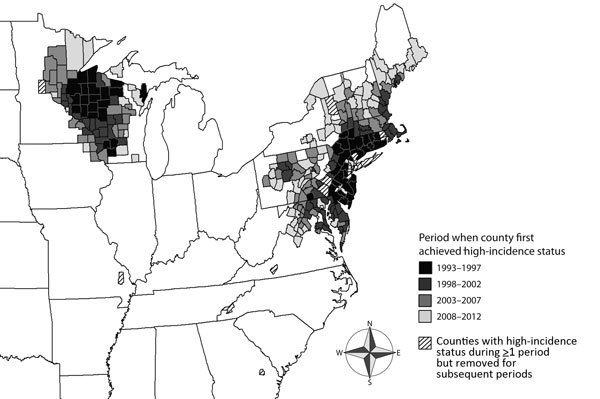
United States counties with high incidence of Lyme disease by the period when they first met the designated high-incidence criteria, 1993–2012. High-incidence counties were defined as those within a spatial cluster of elevated incidence and those with >2 times the number of reported Lyme disease cases as were expected (based on the population at risk).

**Table:** Data for United States counties with high incidence of human Lyme disease during four 5-year periods, 1993–2012*

Location, period	No. counties	Relative risk, range†	Average annual incidence, range‡	No. counties added to high-incidence status	No. counties removed from high- incidence status
Overall					
1993–1997	69	2.3–91.1	10.6–402.7	NA	NA
1998–2002	130	2.0–152.6	12.3–912.9	71	10
2003–2007	197	2.0–101.3	15.0–742.8	72	5
2008–2012	260	2.0–48.6	15.9–381.4	72	9
Northeastern focus					
1993–1997	43	2.3–91.1	10.6–402.7	NA	NA
1998–2002	90	2.0–152.6	12.3–912.9	50	3
2003–2007	130	2.0–101.3	15.0–742.8	45	5
2008–2012	182	2.0–48.6	15.9–381.4	60	8
North-central focus					
1993–1997	22	2.6–41.3	12.1–189.6	NA	NA
1998–2002	40	2.0–35.3	12.4–217.3	21	3
2003–2007	67	2.0–29.8	15.0–222.7	27	0
2008–2012	78	2.1–28.1	16.1–220.7	12	1

*In the first period, 4 counties in the southeastern United States met high-incidence criteria but are not included in the geographic focus–specific data. NA, not applicable. †Relative risk is observed number of cases over a period divided by expected number of cases for the population at risk (*4*). For this analysis, high-incidence counties had a relative risk of >2.0. ‡Incidence is reported cases per 100,000 residents per year; population used was average population at risk during a period (US census data for midpoint of each period).

Over time, the number of counties in the northeastern states identified as having high incidence of Lyme disease increased >320%: from 43 (1993–1997) to 90 (1998–2002) to 130 (2003–2007) to 182 (2008–2012). In the north-central states for the same periods, the number of counties having high incidence increased ≈250%, from 22 to 40 to 67 to 78. In each of the latter periods, a small number of counties previously identified as having high incidence ceased to meet the criteria; however, most remained above the threshold during all periods assessed (Table).

The county geographic center of each major focus was calculated according to Euclidean distances between county centroids by using ArcGIS 9.3 (Environmental Systems Research Institute, Redlands, CA, USA). The center of the high-incidence focus in the northeastern United States generally moved westward and northward, away from the coast of northern New Jersey and into east-central Pennsylvania. In the north-central high-incidence focus, the geographic center remained relatively stable in northwestern Wisconsin, moving northward and southward between adjacent counties over time.

## Conclusions

We describe a simple measure for objectively defining counties having high incidence of Lyme disease. Systematic application of this method to 4 consecutive periods showed geographic expansion of high-risk areas. Despite the substantial increase in the number of counties with high incidence, the limited movement of the geographic centers suggests relatively constant rates of geographic expansion in all accessible directions.

Although risk maps for Lyme disease have been developed on the basis of entomologic measures such as density of and infection prevalence in nymphal *Ixodes scapularis* vector ticks, these measures do not uniformly predict risk of human Lyme disease (*6*,*7*). Prior analyses of temporal trends in human Lyme disease surveillance have not been explicitly spatial or have been conducted by using data from a single state (*8*–*13*).

Surveillance data are subject to several limitations, including changing surveillance case definitions, availability of public health resources for surveillance, variations in surveillance practices, and reporting based on county of residence instead of county of exposure. Nevertheless, in accordance with the purpose of public health surveillance, these data provide valuable information about the magnitude and geographic distribution of areas in the United States where residents are at high risk of acquiring Lyme disease (*5*,*14*).

Four counties in the southeastern United States had high incidence of human Lyme disease during the early years of national surveillance but subsequently had low incidence. This circumstance may reflect improved standardization of diagnostic procedures and a recognition that another condition, southern tick-associated rash illness (also known as STARI), occurs in the region. Patients with this illness have rash similar to that of Lyme disease, but the condition is not caused by *B. burgdorferi* bacteria *(**15**).* The ability to identify these isolated counties shows that our method is not biased toward detecting only counties near other areas with high incidence of Lyme disease.

A true reduction in human risk for Lyme disease or changes in surveillance practices may have influenced the small number of counties meeting high-risk criteria during 1 period but not in subsequent periods. The RR cutoff of 2.0 was arbitrarily chosen to capture counties with not just elevated risk but a substantially higher risk for disease than other counties. The overall pattern of expansion in each period was similar when RR cutoffs of 1.5 and 3.0 were used (data not shown). However, using different RR thresholds to define high incidence changes the number of counties that meet the high-incidence criteria. This variation underscores that risk can be elevated in areas that fail to meet our high-incidence threshold.

Risk for encounters with infected ticks, even within high-incidence counties, is influenced by human behavior and varying landscape characteristics that impact tick abundance and small mammal species composition. Geographic expansion of high-risk areas may occur because of changes in conditions that favor tick survival or because of geographic dispersal of infected ticks by birds and deer to areas where other necessary components already exist to support ongoing transmission. Our results show that geographic expansion of high-risk areas is ongoing, emphasizing the need to identify broadly implementable and effective public health interventions to prevent human Lyme disease.
